# Buzzing Sympathetic Nerves: A New Test to Enhance Anisocoria in Horner's Syndrome

**DOI:** 10.3389/fneur.2019.00107

**Published:** 2019-02-21

**Authors:** Rawan Omary, Christopher J. Bockisch, Klara Landau, Randy H. Kardon, Konrad P. Weber

**Affiliations:** ^1^Department of Ophthalmology, University Hospital Zurich, University of Zurich, Zurich, Switzerland; ^2^Department of Neurology, University Hospital Zurich, University of Zurich, Zurich, Switzerland; ^3^Department of ENT, University Hospital Zurich, University of Zurich, Zurich, Switzerland; ^4^Department of Ophthalmology, University of Iowa and Veterans Medical Center, Iowa City, IA, United States

**Keywords:** Horner's syndrome, pupillometry, sympathetic activation, electrical stimulation, anisocoria, brimonidine

## Abstract

**Introduction:** Patients with suspected Horner's syndrome having equivocal pupil dilation lag and pharmacologic testing may undergo unnecessary MR imaging and work up in the case of false positive pupil test results. Our goal was to increase the diagnostic accuracy of pupillometry by accentuating the inter-ocular asymmetry of sympathetic innervation to the iris dilator with surface electrical stimulation of the median nerve using a standard electromyography machine. We hypothesized that an accentuated difference in sympathetic response between the two eyes would facilitate the diagnosis of Horner's syndrome.

**Methods:** Eighteen patients with pharmacologically proven Horner's syndrome were compared to ten healthy volunteers tested before and after monocular instillation of 0.2% brimonidine tartrate ophthalmic solution to induce pharmacological Horner's syndrome. Pupillary responses were measured with binocular pupillometry in response to sympathetic activation by electrical stimulation of the median nerve in darkness and at various times after extinction of a light stimulus. Sudomotor sympathetic responses from the palm of the stimulated arm were recorded simultaneously.

**Results:** In subjects with Horner's syndrome and pharmacologically induced unilateral sympathetic deficit, electrical stimulation in combination with the extinction of light greatly enhanced the anisocoria during the evoked pupil dilation, while there was no significant increase in anisocoria in healthy subjects. The asymmetry of the sympathetic response was greatest when the electrical stimulus was given 2 s after termination of the light or under constant low light conditions. When given 2 s after termination of light, the electrical stimulation increased the mean anisocoria from 1.0 to 1.2 mm in Horner's syndrome (*p* = 0.01) compared to 0.22–0.26 mm in healthy subjects (*p* = 0.1). In all subjects, the maximal anisocoria induced by the electrical stimulation appeared within a 2 s interval after the stimulus. Correspondingly, the largest change in anisocoria between light and dark without electrical stimulation was seen between 3 and 4 s after light-off. While stronger triple stimulation further enhanced the anisocoria, it was less well tolerated.

**Conclusions:** Electrical stimulation 2 s after light-off greatly enhances the sensitivity of pupillometry for diagnosing Horner's syndrome. This new method may help to rule in or rule out a questionable Horner's syndrome, especially if the results of topical pharmacological testing are inconclusive.

## Introduction

The clinical diagnosis of Horner's syndrome (HS) relies on the classical triad of ipsilateral pupillary miosis, blepharoptosis, and facial anhidrosis, which result from an interruption of the sympathetic innervation to the eye and ocular adnexa. Pupillary dilation lag, which is considered to be the most specific feature of Horner's syndrome, is not routinely used for diagnosis due to the difficulty in detecting it with clinical certainty, leaving pharmacologic testing with cocaine or apraclonidine as the gold standard despite their own limitations of availability, added time to a clinic visit, and correct interpretation. The resulting diagnostic uncertainty in borderline cases often leads to patient anxiety, unnecessary neuroimaging and workup.

Previous attempts to use automated pupillometry for the diagnosis of HS through the detection of pupillary dilation lag have shown very high specificity yet the sensitivity was low (1).

Knowing that unilateral Horner's syndrome occurs due to a sympathetic innervation defect in one eye, we suggest that by delivering a generalized sympathetic stimulation to both eyes, we can cause enhancement of the anisocoria in patients with HS but not in healthy subjects.

General sympathetic activation can be achieved through a painful stimulus (2, 3), such as that caused by an electrical surface stimulation to the median nerve at the wrist using a standard electromyography (EMG) machine. Using automated pupillometry, we look for an increase in the anisocoria and difference in pupil dilation velocity in reaction to the enhanced sympathetic activation, to more sensitively detect a unilateral sympathetic innervation deficit.

Patients with HS can have different degrees of ocular sympathetic deficit, depending on the underlying site of nerve damage or the duration of HS. In order to control for those variables, we treated one eye of healthy subjects with 0.2% brimonidine tartrate ophthalmic solution, which is known to induce a sympathetic block in the eye owing to the alpha-2 adrenergic agonist effect (4, 5), blocking the release of norepinephrine, resulting in a complete pharmacological HS.

With this new method, our goal is to enhance the diagnostic accuracy of pupillometry for the diagnosis of Horner's syndrome with electrically induced sympathetic activation.

## Materials and Methods

### Protocol Approval, Registrations, and Patient Consents

Written informed consent was obtained from all participants, and the protocol was approved by the Zurich cantonal ethics committee, Switzerland (BASEC-Nr. 2016-02151), in accordance with the Helsinki Declaration.

### Participants

Participants were tested at the University Hospital of Zurich between October 2017 and August 2018. Eighteen patients (8 female) with proven Horner's syndrome (HS) (age mean 59 years, range 38–83 years) and 10 healthy volunteers (4 female, age mean 39 years, range 25–52 years) took part in the study. Etiologies for HS included: long-standing and new onset HS of unknown etiology, surgically induced HS, cervical lesions, and internal carotid artery dissection. Inclusion criteria for the patients' group were: older than 18 years, unilateral HS previously confirmed using cocaine eye drops test (6), no past ocular surgery or trauma with residual iris sphincter damage, and no topical or systemic medications that could affect pupillary responses. Healthy subjects' inclusion criteria: older than 18 years, no past pupillary disorders, no past ocular surgery or trauma, no chronic topical or systemic medications use. Exclusion criteria for both groups: the presence of a cardiac pacemaker or defibrillator.

### Study Design

Case-control study.

### Experimental Procedure

A binocular pupillometer (DP-2000, Neuroptics, Irvine, CA, USA) was used to produce light stimuli and record the pupils of all subjects. Electrical stimulation was provided using a standard electromyography (EMG) machine (Nicolet Viking Quest, Natus Medical Incorporated, Pleasanton, CA, USA).

**Figure 1:**
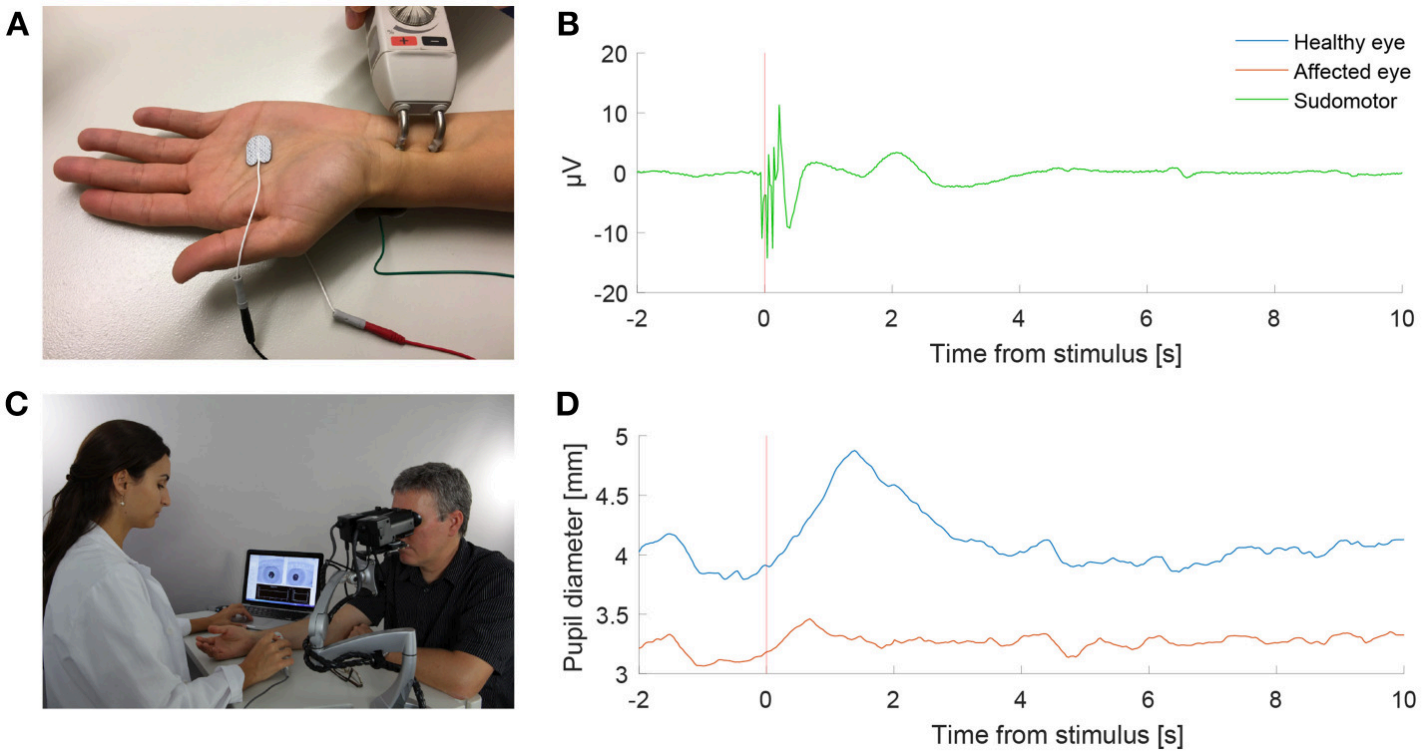
Pupillometry with a “buzz”. **(A)** Electrical sympathetic stimulation (”a buzz”) is delivered to the median nerve using a standard electromyography (EMG) stimulator. Red and black electrodes record sympathetic skin response (SSR). Green electrode is ground. **(B)** Normal SSR recording in a patient with Horner's syndrome. **(C)** Pupil dynamics are recorded simultaneously using automated binocular pupillometry. **(D)** Pupil size over time in a patient with Horner's syndrome as measured with pupillometry, showing the timely synchronized appearance of the increase in anisocoria with the SSR in response to the electrical stimulation. (Photograph in C is published after obtaining a written informed consent from the appearing persons).

With the participant sitting and looking into the eyepieces of the pupilometer, binocular pupillary recording (frame rate 30 Hz) was done for each test paradigm (Figure 1). In a dark and quiet examination room, the participants were asked to look straight ahead into the video cameras for about 1–1.5 min depending on the test paradigm, during which they were asked to close their eyes for 4 s at the end of each repetition to prevent ocular irritation and blinking at critical recording times. An electrical stimulus was delivered using the stimulator of the EMG machine to the median nerve at the wrist (Figure 1A), similar to that used in the sympathetic skin response test (SSR) (7). Conducting gel [Ten20, Weaver and Company, Aurora, USA] for the tip of the stimulator, as well as a grounding sticker electrode [Neuroline Ground, Ambu, Copenhagen, Denmark] at the back of the hand, were used, as in the standard SSR test. The electric current of a single electrical stimulus was chosen to be 55 mA during 0.2 ms, and a triple stimulus was defined as three consecutive single stimuli (12 Hz). The devices were synchronized so that the pupillometer triggered the EMG machine to deliver an electrical stimulus at specified times as programmed for each test paradigm.

The SSR was recorded from electrodes at both sides of the hand (Figure 1A) in 2 patients and 2 healthy volunteers as a reference. SSR represents the potential generated in skin sweat glands in response to sympathetic stimulation. SSR in response to median nerve stimulation was recorded simultaneously to the pupil reaction to compare the timing of the pupillary reflex dilation to the SSR.

Patients with HS received the stimulation to the ipsilateral median nerve (same side as the HS), and healthy volunteers were assigned randomly to receive the electrical stimulation to the right or left median nerve. The purpose of the electric stimulus was to induce a general sympathetic activation through the associated pain, rather than a direct activation of the median nerve (2, 3).

After completing the initial tests, healthy volunteers were treated with 0.2% brimonidine tartrate ophthalmic solution to one eye only, assigned randomly, and the tests were all repeated 45 min after the drop instillation. Brimonidine is an alpha-2 adrenergic agonist, which causes inhibition of norepinephrine secretion, resulting in a sympathetic block to the iris dilator (4, 5), resembling the pupil's state in a complete HS.

### Test Paradigms

Pupil responses were recorded in response to cycles of light and dark, to electrical stimulation during constant low (0.1 log-lux) and high (2.5 log-lux) illumination, and to electrical stimulation at different time points during the cycles of light and dark. Examples of test paradigms are shown in Supplementary Videos 1–5.

Each subject first underwent baseline binocular pupil recordings, without electrical stimulation. The main paradigm consisted of cycles with 4 s of white light-on (3 log-lux) followed by 20 s of darkness, and was repeated at least four times. Pupil responses were also recorded for 17 s of constant light stimulation with levels of 0.1 and 2.5 log lux. Next, the paradigms were repeated with the addition of electrical stimulation. For the cyclic paradigm, the stimulation occurred at different time points (at 0.5 s before, simultaneously with, and 2 s after the termination of light). Only one stimulation was presented per light/dark cycle, and four repetitions of each stimulation time point were included. In the constant light paradigms, the electrical stimulation occurred after 4 s of constant light stimulation.

To avoid an “order bias” which can be caused by response habituation after repeated nerve stimulation (8), the test paradigms with electrical stimulation were performed in a randomized order for each subject, and a 5–10 min break with turning the room light on and engaging the subject in a conversation were taken half way through the experiment.

In order to assess the pupils' reaction to different levels of electrical stimulation, three of the aforementioned test paradigms, namely the electrical stimulus given at 0.5 s before and 2 s after light-off, and the electrical stimulation alone paradigm with 0.1 log-lux light intensity, were repeated with a triple stimulus, performed last in each testing session.

### Data Analysis and Statistics

Videos recorded by the pupillometer were analyzed with custom programs written in MATLAB and the Image Processing and Statistics toolboxes 2016b (The MathWorks Inc., Natick, Massachusetts, United States). Pupils were found by thresholding the image, the pupil edge identified with the MATLAB function “bwboundaries.m,” and an ellipse was fitted to the edge (9). The vertical diameter of the fitted ellipse was used as a measure of pupil size, since this will not change with horizontal eye movements, which seemed more common than vertical eye movements in our paradigms (a fixation target was not present during recording, and subjects were reminded to try and maintain straight-ahead gaze as necessary). Recordings of artificial pupils were used to calibrate the pupilometer and to convert pupil diameter from pixels to millimeters. The measured values were removed (usually owing to full or partial blinks or large eye movements) if the fitted ellipse deviated too far from a circle (ratio of major to minor axes >1.3), if the pupil diameter was <1.25 or >8 mm, if pupil constriction/dilation velocity exceeded 10 mm/s, or the duration of the eyes being open was <0.5 s. Entire trials were rejected if more than one third of the data of either eye was lost.

Pupillary dilation lag was defined as the change in anisocoria (difference in pupil diameter) between 5 and 15 s after the light stimulus was removed (1). For patients, we always measured anisocoria as the healthy pupil size minus the affected pupil size; for healthy subjects we took the absolute value of the difference in pupil size. Pilot experiments showed that the effect of electrical stimulation was limited to the 2 s period following stimulation, so we defined the electrically-induced anisocoria as the maximum anisocoria during this period. To determine the effect of electrical stimulation during trials where there was a changing light stimulus, compared to a baseline measure of ansiocoria, we also calculated the maximum anisocoria during the 2 s interval on the equivalent trials without electrical stimulation. For constant light-on trials, we used the anisocoria just prior to electrical stimulation as the baseline.

To determine the optimal time point for measuring anisocoria that differentiates patients from healthy subjects without electrical stimulation, we calculated the relative change in anisocoria between light and dark over 1 s for each second after light-off. The relative change in anisocoria was defined as the median anisocoria during that time interval minus the anisocoria at the end of the light-on period. The area under the receiver operating characteristic curve (AUC) was then calculated at each time interval.

To evaluate the effect of electrical stimulation, we performed paired *t*-tests with Holm's correction for multiple comparisons within each subject group. In order to evaluate which electrical stimulation condition produced the most consistent differences from non-electrical stimulation conditions, we calculated the mean differences to z-scores (mean difference/standard deviation of the pair differences). Larger z-scores thus indicated that electrical stimulation produced a larger consistent effect, and could be produced either by a larger mean difference, or by smaller variability.

## Results

We measured 18 patients with Horner's syndrome (HS) and 10 healthy volunteers before and after monocular instillation of brimonidine drops. Using automated pupillometry, we compared the anisocoria and difference in pupillary dilation velocity with and without electrical stimulation.

**Figure 2:**
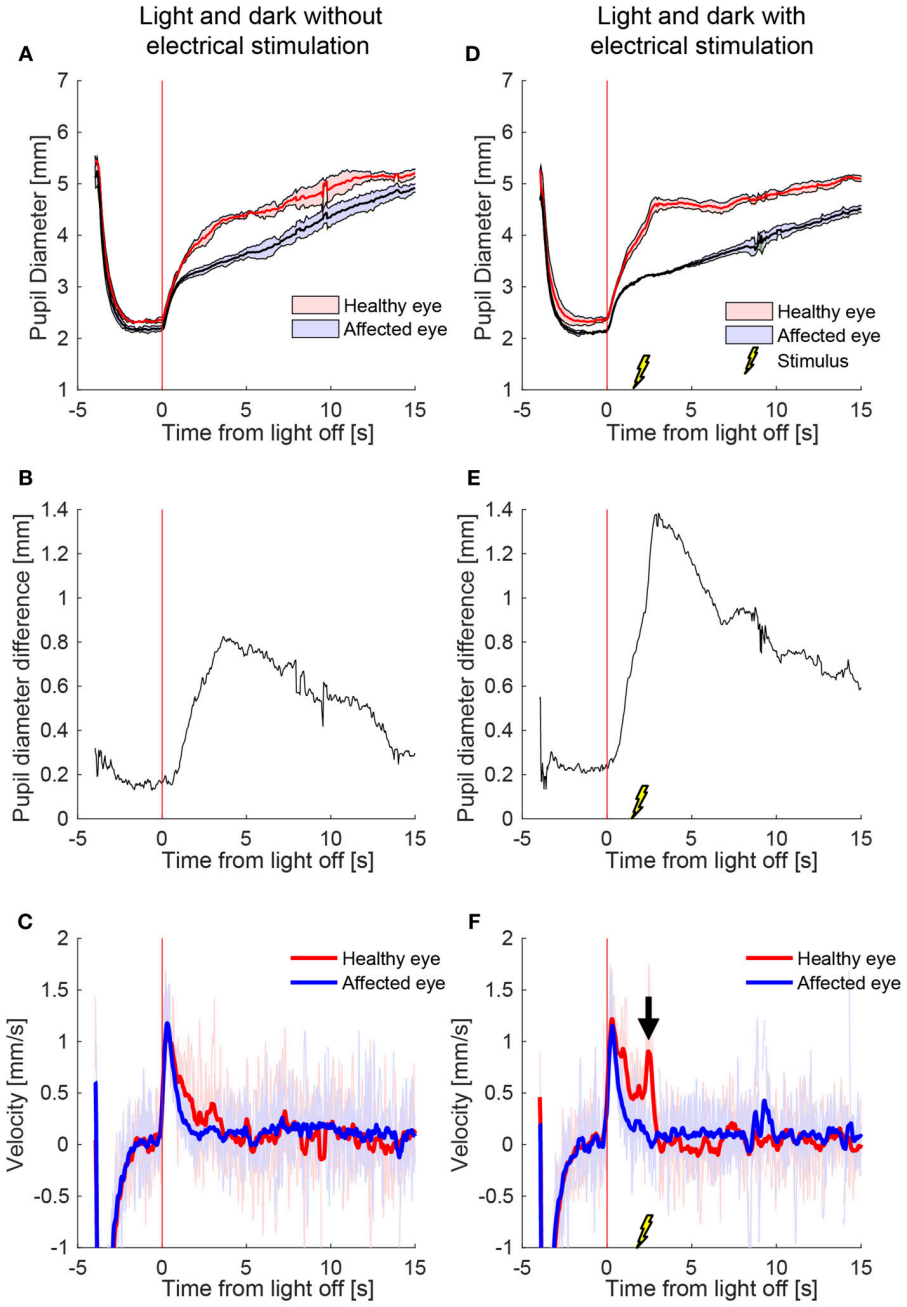
The effect of electrical stimulation (“buzzing”) in a patient with Horner's syndrome. The left column shows the pupil response to a cycle of light and dark alone, and the right column shows the response with the addition of electrical stimulation delivered 2 s after light-off **(A)** Pupils size over time in a patient with HS, as seen on pupillometry with light/dark alone. The dark lines are the means of 5 trials, and the shaded region represents ±1 standard deviation. Time zero indicates extinction of the light, after which the difference between the pupils starts to increase. **(B)** The difference in mean pupil size, showing a maximal difference of about 0.8 mm, 4 s after the light is turned off **(C)** Pupil dilation velocity over time for the same test: a difference is noted in the early dilation phase between the healthy and affected pupils. **(D–F)** When an electrical stimulation is introduced at 2 s after light-off, a clear increase in anisocoria **(D,E)** and second dilation velocity peak (arrow) of the healthy eye **(F)** appear.

Figure 2 shows a representative example of the pupil light responses of a patient with left HS without (left column) and with (right column) electrical stimulation delivered 2 s after the extinction of light. The healthy right pupil (red) of this patient dilated normally in the dark, expanding from a little more than 2 mm in diameter in the light to about 5 mm after 15 s (Figure 2A). The affected left pupil (blue), however, dilated similarly in the first second, but then dilation slowed, with a maximum anisocoria of about 0.8 mm appearing about 4 s after light off (Figure 2B). In Figure 2C the dilation velocity graph for each pupil shows that the right healthy pupil (red) has a larger dilation velocity in the first 3 s after light off. Electrical stimulation 2 s after light off increased the dilation in the healthy right pupil, but had no discernable effect in the affected left pupil (Figure 2D), resulting in an increase in maximum anisocoria to about 1.4 mm (Figure 2E) as well as an increase in the difference in pupillary dilation velocity between the healthy and affected pupil (Figure 2F, arrow).

**Figure 3:**
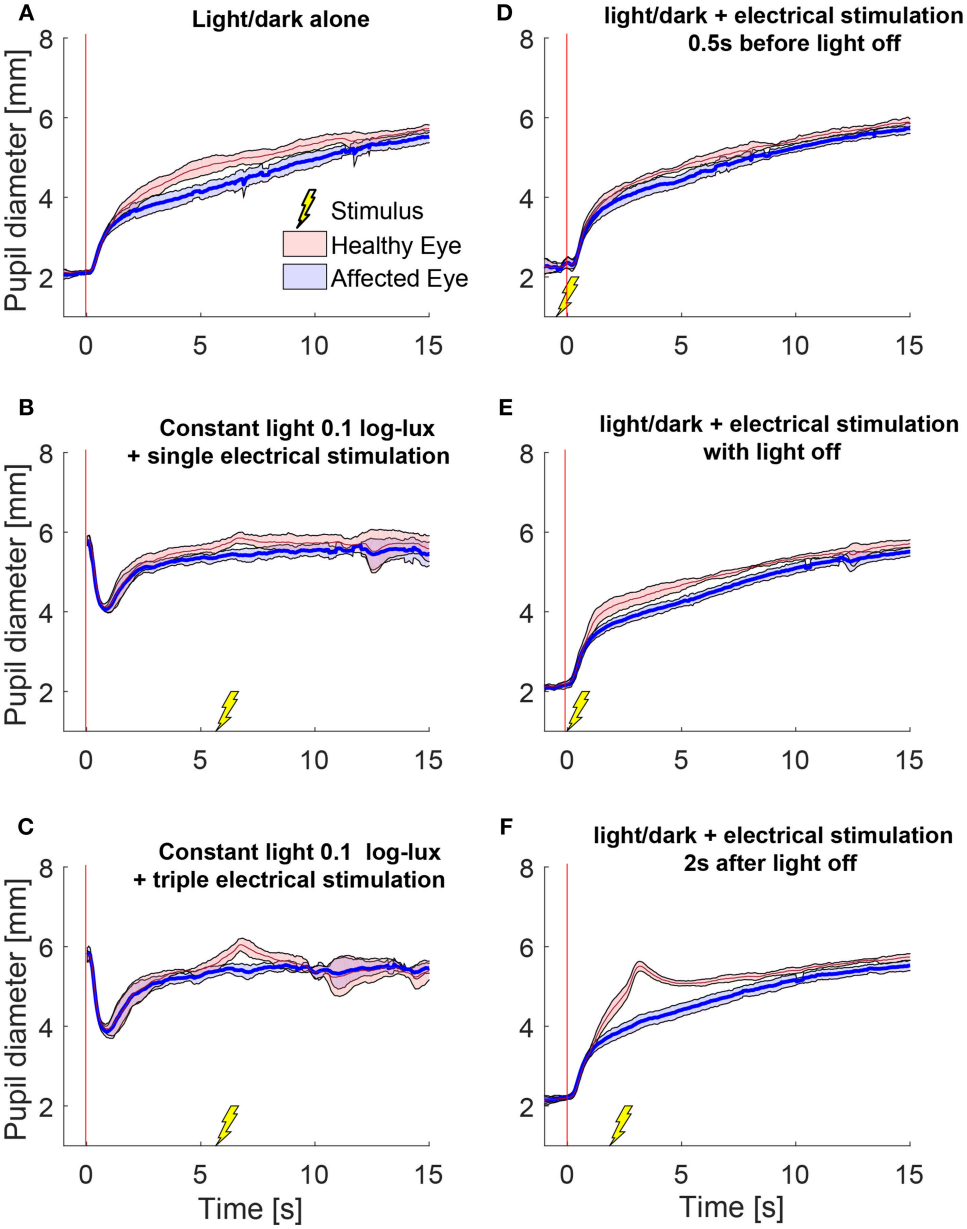
Pupillary responses to light/dark alone, electrical stimulation alone, and the combination of the two in a patient with HS. All traces are the means (thick lines) ±1 standard deviation (shaded areas). **(A)** Pupillometry with light and dark alone (no electrical stimulation). **(B)** Electrical stimulation alone during constant low light with single stimulus and **(C)** with triple stimulus. After the initial constriction in response to the low light, pupils are allowed to reach a steady state for 5 s before the electrical stimulation is given at 6 s. **(D–F)** Combined test paradigms: electrical stimulation during cycle of light/dark at minus 0.5 s **(D)**, 0 s **(E)**, and 2 s **(F)** from-light-off.

We also tested the effect of electrical stimulation at different times relative to light-off. Figure 3 shows the average responses of one patient during different test paradigms. A single electrical stimulus in low light (0.1 log-lux) (Figure 3B) produced a small change in anisocoria, whereas the triple stimulus during similar light conditions produced a noticeable dilation response in the healthy eye (Figure 3C) causing a larger increase in anisocoria. When electrical stimulation occurred 0.5 s before (Figure 3D) or with (Figure 3E) the light extinction, the effect of the stimulus was smaller and no discernable increase in anisocoria was noticed. Electrical stimulation at 2 s after light off (Figure 3F) produced the largest increase in anisocoria as compared to the other test paradigms.

**Figure 4:**
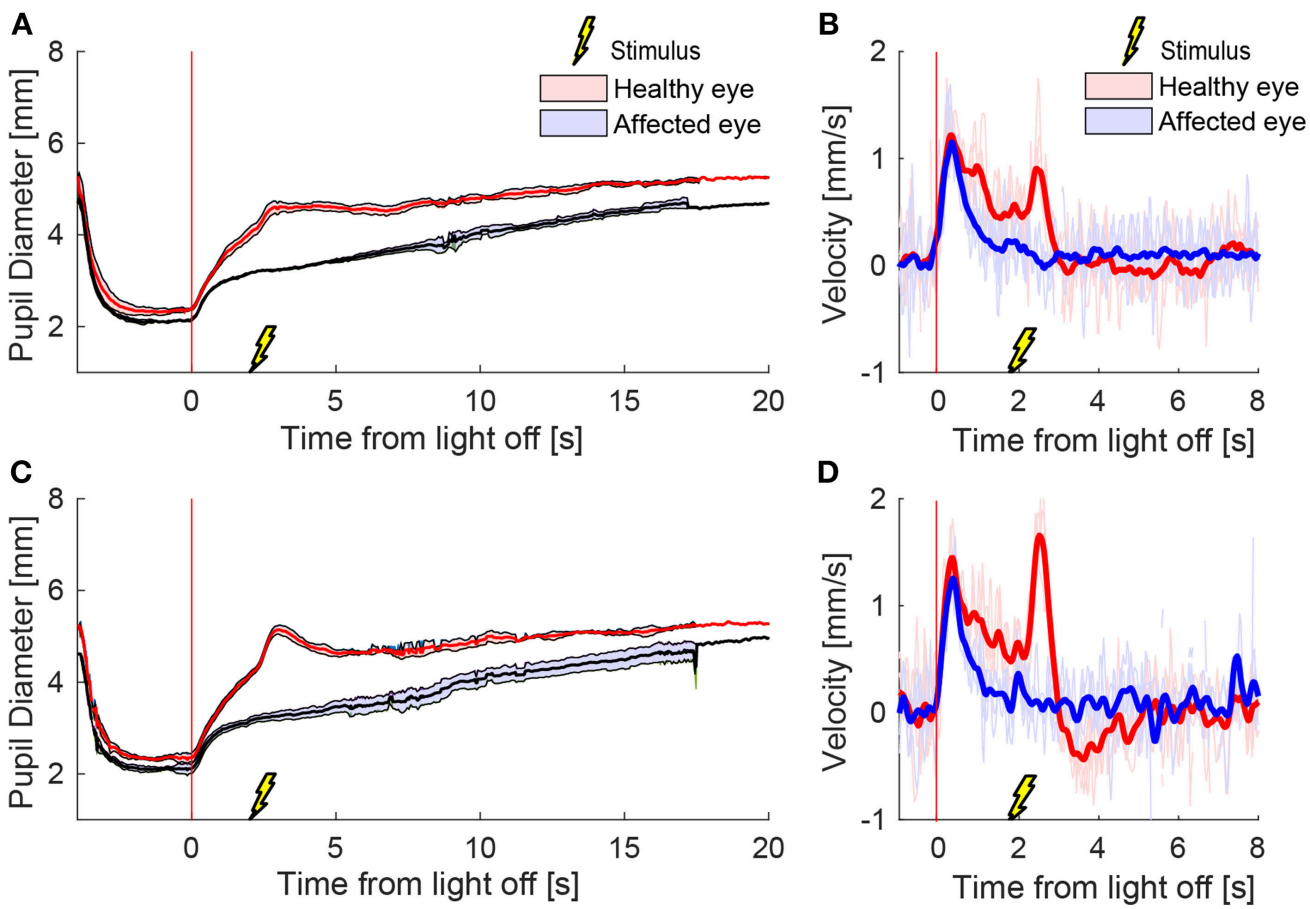
Different stimulation intensities. In a patient with HS, a single electrical stimulus (55 mA, 0.2 ms) causes an increase in difference between the pupils in size **(A)** and dilation velocity **(B)**. A triple stimulus (3x single stimulus, 12 Hz) causes a larger difference between the pupils **(C,D)** compared to the single stimulus.

To test the effect of stimulus intensity, we applied three electrical stimuli in quick succession at 12 Hz (triple stimulus). Figure 4 shows an example of mean pupil responses in a patient with HS to the triple stimulation compared to the single stimulation during a cycle of light and dark. The increase in both the anisocoria (Figure 4A) and difference in pupil dilation velocity (Figure 4B) produced by the single stimulus were further increased in the same patient when a triple stimulus was given (Figures 4C,D).

**Figure 5:**
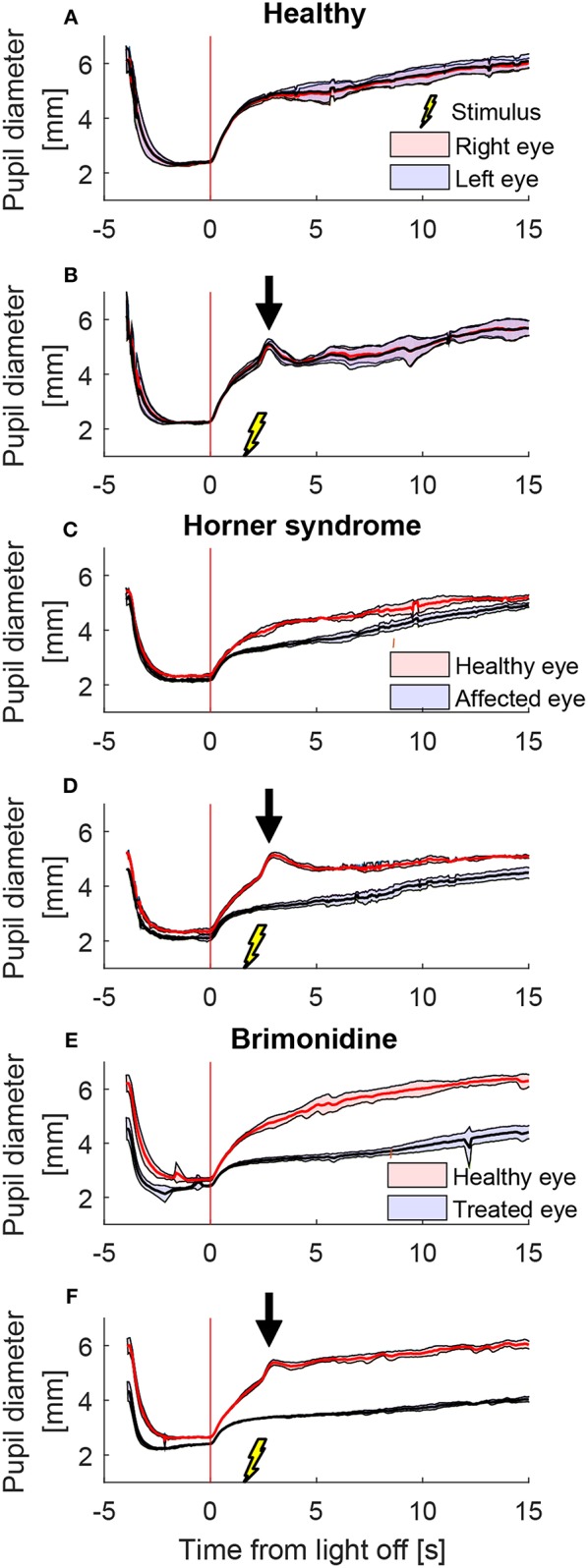
Comparison of pupillary response with and without electrical stimulus. All traces are the means (thick lines) ±1 standard deviation (shaded areas) of 4–5 trials (trials could be lost due to blinks) **(A)** a cycle of light and dark alone in a healthy subject, shows no anisocoria. **(B)** Adding an electrical stimulus 2 s after light-off in a light and dark cycle results in a second dilation peak (arrow) yet does not provoke a difference between the pupils as compared to light and dark alone. In a patient with Horner's syndrome **(C,D)** as well as in a healthy subject treated with brimonidine **(E,F)**, electrical stimulation results in an increase in the anisocoria as compared to a similar test paradigm of light and dark alone (arrows).

A representative example of mean pupil responses to electrical stimulation (triple stimulus condition) compared to the condition without electrical stimulation is shown in Figure 5 in a healthy subject (Figures 5A,B), a patient with HS (Figures 5C,D), and a healthy subject treated with brimonidine (Figures 5E,F). The triple-stimulus produced a prominent increase in anisocoria in the HS patient and the subject with brimonidine for a couple of seconds, whereas no increase or induction of anisocoria was seen in the healthy subject as a result of the stimulation.

**Figure 6:**
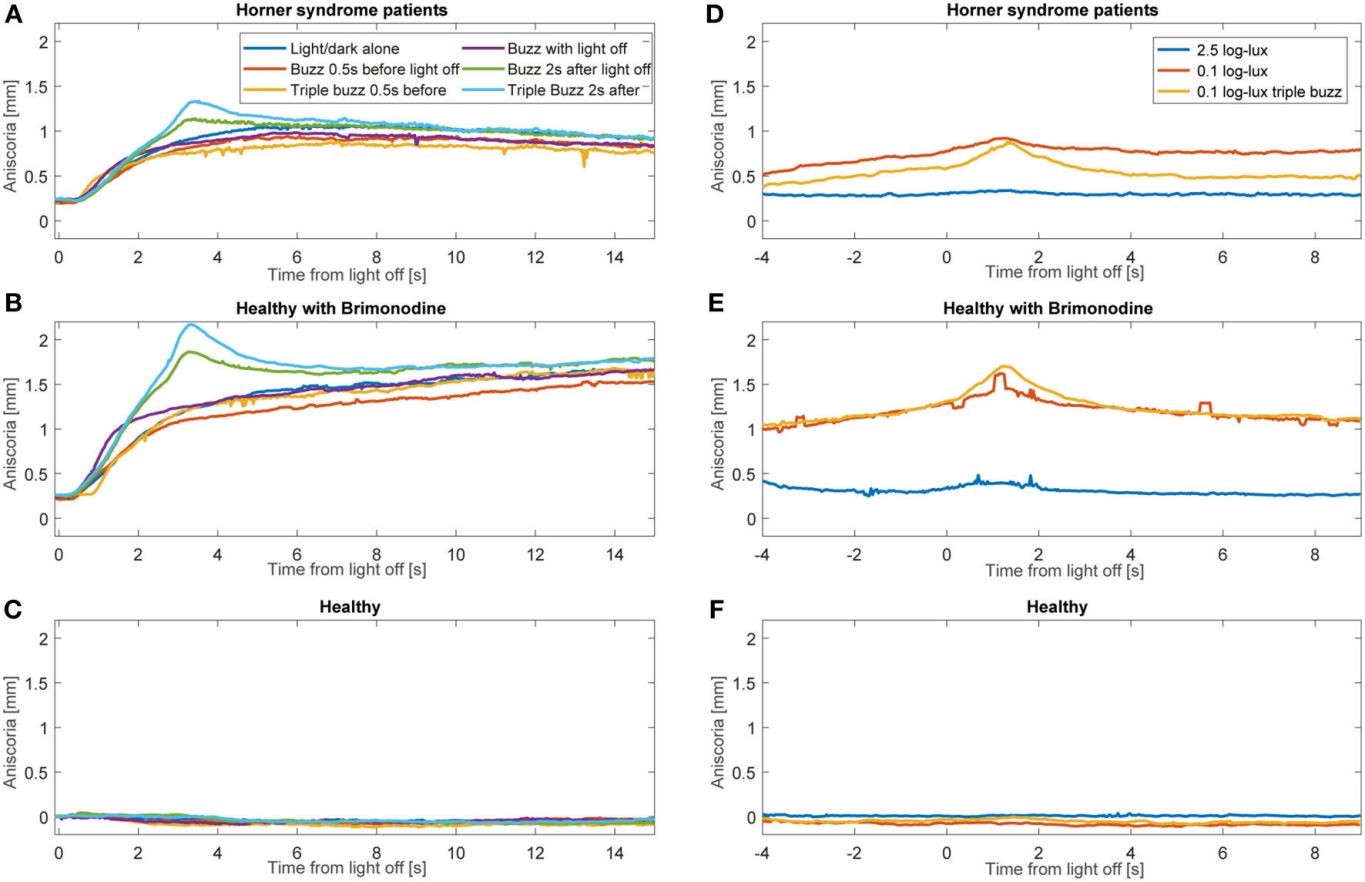
Average anisocoria traces for each group. Graphs **(A–C)** show the average anisocoria resulting from different conditions during cycles of light and dark. In the patient group **(A)** and the brimonidine group **(B)** we notice a larger transient increase in anisocoria in response to single and triple electrical stimulation at 2 s after light goes off as compared to the test paradigm without electrical stimulation. Electrical stimulation at the other times did not produce as discernable increase. **(C)** In the healthy group, no anisocoria was measured or induced during all the test paradigms. Graphs **(D–F)** show the average anisocoria resulting from different conditions during constant light-on conditions. Single electrical stimulation during constant light on of 0.1 log-lux intensity produced an increase in anisocoria in both the patients group **(A)** and brimonidine group **(B)** but not in the healthy group **(C)**. Triple stimulation during similar light conditions produced a larger increase in anisocoria for the patient **(A)** and brimonidine **(B)** groups. Stimulation during constant light on of 2.5 log-lux intensity did not increase the anisocoria in any of the 3 groups.

Average anisocoria traces for each of the groups are shown in Figure 6 (Figures 6A–C during light and dark cycles, Figures 6D–F in constant light-on conditions). As expected, there was little measured anisocoria in healthy subjects, whereas substantial increases in anisocoria were noted in HS patients and healthy subjects with brimonidine in response to electrical stimulation as compared to without. Note that in our patients during cycles of light and dark without electrical stimulation, the average anisocoria was largely constant in the period from 5 s after light off (1.0 mm) to the end of the trial (0.9 mm) (Figure 6A).

**Figure 7:**
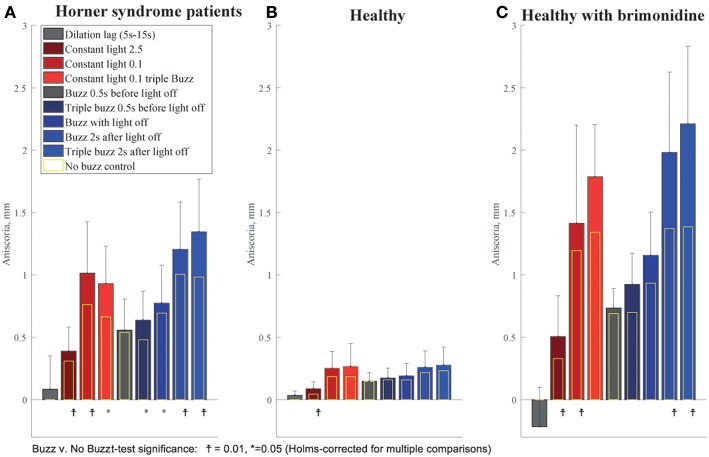
Summary of buzzing effect. Each bar shows the measured anisocoria in response to electrical stimulation compared to the baseline anisocoria (yellow bars) for each matched condition with no electrical stimulation. **(A)** In Horner's syndrome patients, electrical stimulation produced a significant increase in aniscoria in all conditions except for the single stimulus given 0.5 s before light off. **(B)** Except for the constant bright light condition (2.5 log-lux), healthy subjects did not show any significant increase in anisocoria in the test paradigms as compared to parallel no electrical stimulation paradigms. **(C)** Subjects treated with brimonidine showed similar reaction patterns to electrical stimulation as seen in HS group, yet with larger increases in anisocoria.

We observed that the effect of electrical stimulation was generally confined to 2 s after the stimulus. Therefore, we assessed the effect of the electrical stimulation by measuring the maximum anisocoria within 2 s after electrical stimulation, and compared it to the anisocoria during the same time interval in the conditions without electrical stimulation. Figure 7 shows the measured anisocoria in each condition after electrical stimulation (color bars), along with the associated condition without electrical stimulation for each test paradigm (yellow bars) for comparison. Figure 7 also shows a measurement of dilation lag as it was previously defined in literature (1, 10) as the change in anisocoria from 5 to 15 s after light off. Of note is that none of our HS patients or brimonidine subjects had an average dilation lag of 0.4 mm or larger with these parameters. In subjects treated with brimonidine the anisocoria slightly increased over time after light off, giving a negative result when calculating the dilation lag using this method.

For HS patients, electrical stimulation produced a significant increase in anisocoria in all test paradigms except for the single electrical stimulus given 0.5 s before light off during a cycle of light and dark (Figure 7A). The largest increase in anisocoria was found when electrical stimulation occurred 2 s after light off (mean = 1.3 mm, standard deviation (*SD*) = 0.4, *p* < 0.01 for difference from baseline *t*-test) for triple stimulus, and 1.2 mm for single stimulus (*SD* = 0.4, *p* < 0.0001) as compared to 1 mm (*SD* = 0.41, *p* < 0.0001) without stimulus. Electrical stimulation also significantly increased the anisocoria in the constant light condition, particularly with the lower light intensity of 0.1 log-lux. Subjects treated with brimonidine showed the same pattern of results as the HS, though the amount of anisocoria was higher. Healthy subjects did not, in general, show an increase in anisocoria with electrical stimulation (because we took the absolute difference in pupil size for healthy subjects, any change in anisocoria was likely just an increase in variability). Overall, the increase in anisocoria for the healthy group in response to a single and triple electrical stimuli given 2 s after light of was similar and equal to 0.04 (*p* > 0.1). Within the healthy group, three subjects had some physiological anisocoria (mean 0.3 mm). The mean increase in anisocoria for those subjects in response to a single and triple electrical stimuli 2 s after light-off was 0.07 and 0.09 mm, respectively, and for subjects without physiological anisocoria was 0.03 and 0.015 mm, respectively (*p* = 0.5 and 0.42).

**Figure 8:**
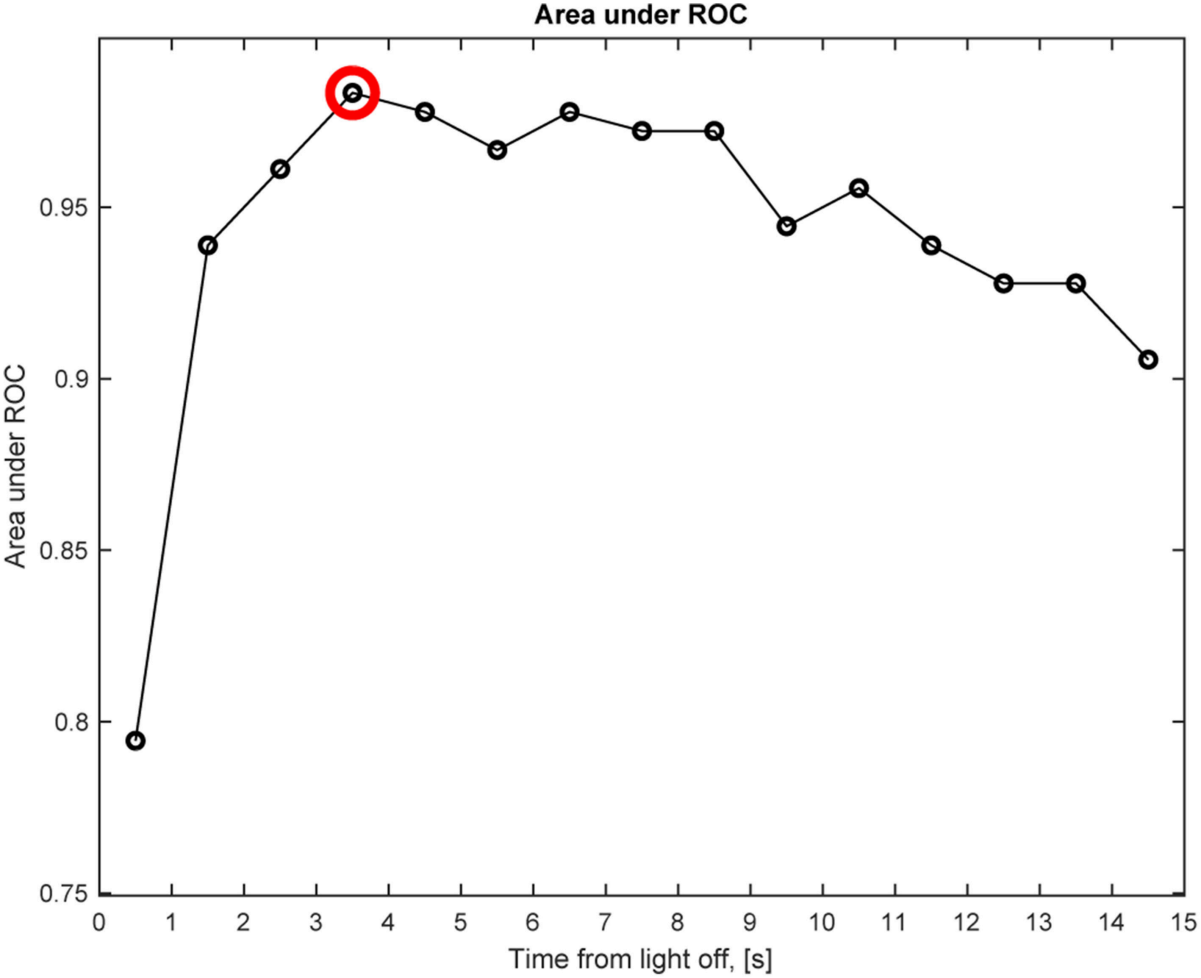
Anisocoria in Horner's syndrome without electrical stimulation. This graph shows the area under the receiver operating characteristic (ROC) curve (AUC) for the anisocoria at each time interval of 1 s after light-off relative to the anisocoria at the end of the light-on period, presented at the matching time interval, e.g., a circle at 0.5 represents the time interval of 0–1 s. The largest AUC occurs at the 3–4 s interval (red circle), indicating that the largest relative change in anisocoria that helps differentiating Horner's syndrome patients from healthy subjects occurs at the 3–4 s interval after light-off.

We also determined the best time point after light off at which the change in anisocoria, relative to light on, best differentiated HS from healthy subjects based on pupillometry without electrical stimulation. The time interval after light termination which gave the greatest area under the receiver operating characteristic curve (AUC) and the best discrimination between patients with HS and healthy subjects, was 3–4 s after light-off, with an AUC of 0.98 (Figure 8). For this time interval, the best discriminating criterion (cut-off value for relative change in anisocoria) was 0.4 mm. Note, however, that AUC was >0.97 for all time intervals between 3 and 8 s, and was above 0.9 for all time intervals except the first second after light off. The AUC for pupillary dilation lag (change in anisocoria from 5 to 15 s after light-off) was only 0.5, and the AUC for all test paradigms with electrical stimulation was more than 0.97.

To determine which of the electrical stimulation conditions gave the largest consistent change in anisocoria, thus taking within-subject variability into account, we converted the differences (electrical stimulation minus no stimulation) into standardized z-scores. The largest z-score was given by the single electrical stimulus under 0.1 log-lux constant light condition (*z* = 2.6). For the light and dark cycles, the triple stimulus given 2 s after light off condition (*z* = 1.1) was best, though just slightly larger than the single stimulus 2 s after light off condition.

## Discussion

### Summary of Results

We found that electrical stimulation increases the anisocoria and difference in dilation velocity between the pupils of subjects with unilateral ocular sympathetic deficit both in Horner's syndrome (HS) and a pharmacologically induced sympathetic block. In contrast, no significant anisocoria was induced in healthy volunteers in response to the electrical stimulation including those with slight physiological anisocoria. The combination of electrical stimulation with cycles of light and dark produced the largest and most consistent enhancement in anisocoria, as compared to either one alone. Pupillary dilation lag with its previous definition is not helpful as a diagnostic measure for HS. Electrical stimulation 2 s after light off and stimulation during constant low light of 0.1 log-lux produced the largest increase in anisocoria compared to the other test paradigms. Electrical stimulation alone caused a larger increase in low light conditions (0.1 log-lux) than in high light condition (2 log-lux). Higher electrical stimulation intensities (triple stimulus) produced a larger increase in anisocoria in HS and pharmacological HS groups as compared to a single stimulus, yet was less well tolerated.

### Previous and Current Tests

The current gold standard for the diagnosis of HS using pharmacologic eye drops testing with either cocaine or apraclonidine, carries several disadvantages, including limited availability of cocaine, longer test duration, and possible false-positive and false negative results (6, 11–14).

Pupillary dilation in response to sympathetic stimulation in the form of auditory stimulus in healthy subjects as well as an increase in anisocoria in subjects with unilateral pharmacological ocular sympathetic block (15), and in patients with HS (16) has been described. In neither study, however, were those stimuli clinically implemented.

The detection of a pupillary dilation lag using pupillometry in patients with HS has been defined as the difference in anisocoria between 5 and 15 s after extinction of the light, and regarded positive when the value is equal to or more than 0.4 mm (1). By this definition, dilation lag had very high specificity for HS yet relatively low sensitivity (48%). In addition, dilation lag was found to be only intermittently present and not consistent from one test to the next in patients with HS, making it unreliable as a clinical diagnostic test (10). In our HS patient group, a positive dilation lag according to this definition was also found in some patients in single cycles of light and dark, yet when averaging the 4–5 test repeats performed for each subject, this value was <0.4 mm for all subjects, thus considered negative. Therefore, our results are in agreement with the previous findings about the intermittent nature of dilation lag in this patient group. This encouraged us to improve the diagnostic accuracy for HS with new pupillometry paradigms and better measurement algorithms.

### The Procedure

Automated binocular pupillometry is a short and easy test to perform, and patient cooperation required is minimal. Electrical nerve stimulation is routinely used in neurology in the sympathetic skin response test (SSR) test among others, and its safety has been long established (17). The synchronization of the EMG machine to the pupillometer facilitates the precise timing of the electrical stimulation during cycles of light and dark. Both the pupillometer and the EMG machines are portable, making it possible to test immobile patients at the bedside.

### Pupillometry With a “Buzz”

All HS patients exhibited anisocoria on pupillometry with cycles of light and dark alone, which significantly increased with the addition of an electrical stimulation. Electrical stimulation before the termination of light (0.5 s before light off) as well as simultaneously with light off produced a smaller enhancement of anisocoria when compared to stimulation at 2 s after light off. This smaller response is likely due to the parasympathetic tone induced by the light stimulus (18), which is no longer present 2 s after the light goes off. At 2 s after light off the pupils are close to the secondary dilation phase which is in the larger part due to sympathetic activity (18) resulting from the termination of light. The addition of the electrical stimulation during this phase seemed to produce the best synergistic sympathetic response between the termination of light and electrical stimulation, resulting in a significant increase in anisocoria. Similarly, electrical stimulation alone (during constant light) produced a larger increase in anisocoria with low background illumination than with higher illumination, which can as well be correlated with the higher parasympathetic tone induced by brighter light.

The triple electrical stimulation resulted in a larger increase in anisocoria compared to similar test paradigms with a single stimulus. The higher the intensity of the stimulus, the larger the increase in anisocoria both in patients and in the brimonidine group. Similar effects of stimulus intensity were demonstrated by Hirano et al. (15) where they showed that louder auditory stimuli produced larger pupillary dilation in healthy pupils.

Using pupillometry without electrical stimulation, we found that the biggest difference in relative anisocoria between patients with HS and healthy subjects occurred when comparing the anisocoria at 3–4 s after light off to that at the end of the light-on period. Using those time points, a 0.4 mm cut-off was found to be the upper limit of relative anisocoria in our normal subjects. This finding is also important since it confirms that the optimal test paradigm for electrical stimulation was where the stimulus was given 2 s after light off, compared to the best measurement of HS found on pupillometry without electrical stimulation.

Patients showed good tolerance to the single electrical stimulation, but tolerance to the triple stimulus was variable. Seven out of eighteen patients declined trying the triple stimulus simply due to the idea of a stronger stimulus, in spite of reporting no pain and minimal discomfort with the single electrical stimulus. Of the 11 patients who agreed to the triple stimulus, 2 patients found it intolerable and stopped, the other 9 completed the experiment and reported it to be “less pleasant” than the lower (single) stimulation, yet still tolerable. Of note, during the experiment the subjects underwent multiple stimulations during all the different test paradigms, which may have influenced the tolerance of patients and increased the discomfort. In a clinical setting, however, only few repetitions would be needed for making the diagnosis of HS, which might lead to less discomfort and higher tolerance for the electrical stimulation. In addition, of the healthy subjects group, all 10 subjects completed the experiment with the triple stimulus twice (before and after brimonidine drops), with only one subject reporting considerable discomfort.

## Summary

In a clinical setting, physicians are facing the challenge of ruling out HS in borderline cases, especially when pharmacological test results are equivocal. We found that compared to pupillometry alone, pupillometry with electrical stimulation 2 s after light off results in an increase of anisocoria in subjects with ocular sympathetic deficit, but not in healthy subjects, which may help distinguish healthy from HS patients.

We found that all patients with HS as well as subjects with brimonidine demonstrated an increase in anisocoria in response to electrical sympathetic stimulation as compared to similar test paradigms without stimulation. The increase in anisocoria in the brimonidine group was larger and more consistent than in HS group, which can be attributed to the complete nature of the sympathetic block induced by brimonidine, compared to the possibly partial sympathetic deficit in patients with HS. This could be due to the different degrees of nerve damage in different HS patients depending on the mechanism and duration of the nerve injury. Healthy subjects demonstrated no significant increase of anisocoria in response to electrical stimulation in contrast to HS patients or brimonidine treated subject on similar test paradigms.

## Limitations

Due to the relatively small number of subjects in this study, it was not possible to assess the effect of different HS etiologies and durations on the responses to electrical stimulation. The reduced tolerability of patients to the triple stimulus resulted in fewer subjects undergoing such test paradigms. In the current study, we only had three patients with some physiological anisocoria, a topic that we plan to address in a follow-up study.

## Conclusions

In this “proof of concept” study, we showed that patients with HS as well as pharmacological ocular sympathetic block demonstrate a significant increase in anisocoria in response to electrical stimulation as measured with binocular pupillometry, while healthy subjects demonstrated no significant anisocoria, reflecting that electrical stimulation may help increase the diagnostic accuracy of pupillometry for HS in a clinical setting.

## Author Contributions

RO: study design, data collection, data analysis, and paper writing; CB: data analysis, statistical analysis, and paper writing; KL: paper revision; RK: study design, paper revision; KW: study design, data analysis, and paper writing.

### Conflict of Interest Statement

The authors declare that the research was conducted in the absence of any commercial or financial relationships that could be construed as a potential conflict of interest.

## Supplementary Material

Supplementary Video 1 Pupillometry without electrical stimulation in a patient with left Horner's syndrome. 5 cycles of light and dark alone without electrical stimulation.

Supplementary Video 2 Pupillometry with electrical stimulation in a patient with left Horner's syndrome. A single electrical stimulation is delivered during constant low light-on (0.1 log-lux).

Supplementary Video 3 Pupillometry with electrical stimulation in a patient with left Horner's syndrome. A triple electrical stimulation is delivered during constant low light-on (0.1 log-lux).

Supplementary Video 4 Pupillometry with electrical stimulation in a patient with left Horner's syndrome. A single electrical stimulation is delivered during each cycle of light and dark, 2 seconds after the termination of light.

Supplementary Video 5 Pupillometry with electrical stimulation in a patient with left Horner's syndrome. A triple electrical stimulation is delivered during each cycle of light and dark, 2 seconds after the termination of light.
